# Regional-level estimation of expected years of life lost attributable to overweight and obesity among Mexican adults

**DOI:** 10.3402/gha.v9.31642

**Published:** 2016-09-06

**Authors:** Efrén Murillo-Zamora, Raúl García-Ceballos, Iván Delgado-Enciso, Raquel Garza-Guajardo, Oralia Barboza-Quintana, Irám P. Rodríguez-Sánchez, Oliver Mendoza-Cano

**Affiliations:** 1 Unidad de Medicina Familiar No. 19, Instituto Mexicano del Seguro Social, Colima, México; 2 Coordinación de Vigilancia Epidemiológica, Servicios de Salud del Estado de Colima, Colima, México; 3 Facultad de Medicina, Universidad de Colima, Colima, México; 4 Instituto Estatal de Cancerología, Servicios de Salud del Estado de Colima, Colima, México; 5 Universidad Autónoma de Nuevo León, Facultad de Medicina, Hospital Universitario “José Eleuterio González”, Servicio de Anatomía Patológica y Citopatología, Monterrey, Nuevo León, México; 6 Universidad Autónoma de Nuevo León, Facultad de Medicina, Hospital Universitario “José Eleuterio González”, Departamento de Genética, Monterrey, Nuevo León, México; 7 Facultad de Ingeniería Civil, Universidad de Colima, Colima, México; 8 Center for Health and the Global Environment, Harvard T.H. Chan School of Public Health, Boston, MA, USA

**Keywords:** obesity, overweight, risk factor, mortality

## Abstract

**Background:**

Excess body weight has become a major public health problem worldwide, and the burden of overweight and obesity was calculated in this work from a health economics perspective.

**Objective:**

To estimate the burden of disease attributable to overweight and obesity among males and females aged 20 years and older using years of life lost (YLL) and age-standardized YLL rates (ASYLL), and to rank the leading causes of premature death.

**Design:**

A cross-sectional study took place (2010–2014) and 6,054 deaths were analyzed. Thirteen basic causes of death associated with overweight or obesity were included. The population attributable fraction (PAF), YLL, and ASYLL were calculated.

**Results:**

The overall burden attributable to overweight and obesity was 36,087 YLL, and the estimated ASYLL per 10,000 persons was 1,098 and 1,029 in males and females, respectively. Type 2 diabetes mellitus was the main cause of premature death (males, 968 ASYLL; females, 772 ASYLL).

**Conclusions:**

Overweight and obesity are major risk factors of chronic diseases that are main causes of premature death in the study population. Strategies for preventing overweight and obesity may decrease the incidence and mortality associated with these non-communicable diseases. ASYLL seems to be an indicator that is particularly well adapted to decision-making in public health.

## Introduction

Overweight (body mass index, BMI, 25–29.9 kg/m^2^) and obesity (BMI ≥30 kg/m^2^) are public health problems because of the substantial burden they represent for health systems in terms of health care and related costs (1, 2). In Mexico, among adults aged 20 years and older, the prevalence of overweight and obesity is 32.4 and 38.8%, respectively (3, 4). Between 1988 and 2006, an average annual increase of 2% in the prevalence of individuals with BMI ≥30 has been documented in Mexican adults, and this increase is the highest recorded worldwide (5).

A high BMI is a major risk factor for type 2 diabetes mellitus, cardiovascular diseases, and other chronic non-communicable diseases associated with a high mortality and disability (2). The excess body weight is also associated with an increased risk of malignant tumors (6).

Worldwide, according to the Global Burden of Diseases (GBD) study, in 2010 exposure to overweight and obesity was estimated to cause 3.4 million deaths and 4% of overall years of life lost (YLL) (7). The GBD methodology has been used at country-level to obtain local measurements of interest (8). In our knowledge, there are not published studies regarding regional (i.e. at state-level) estimation of burden of disease attributable to overweight or obesity in Mexico. From a public health perspective, and in order to minimize the impact of overweight and obesity-related mortality through the implementation of health policies, the accurate measurement of the burden of disease is fundamental (8).

**Fig. 1:**
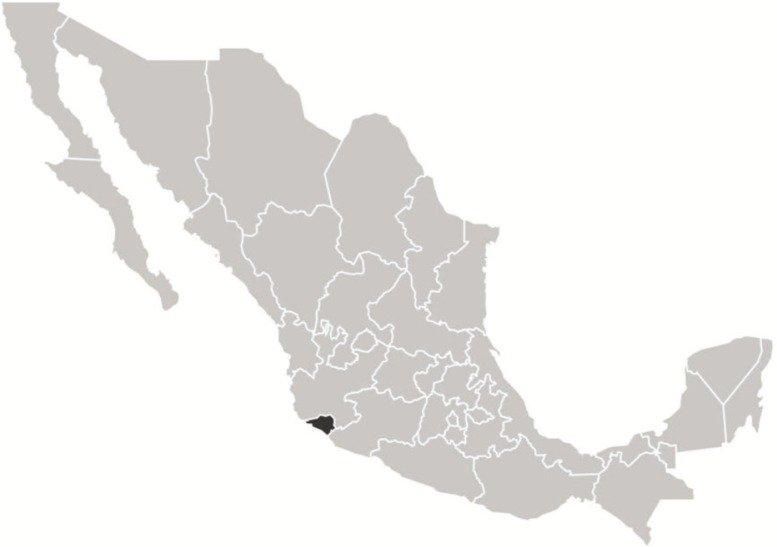
Map of Mexico showing the location of Colima State.

The age-standardized expected YLL rates (ASYLL) are a useful analytical tool to identify and prioritize causes of premature death (9). In comparison with potential YLL (PYLL), which result from the arithmetic subtraction of age at death to a determined cut-off (10), ASYLL incorporate strategies used in cost-effectiveness analysis and are particularly important from a public health perspective because they measure potentially preventable deaths (11). The state of Colima, located on the western coast of Mexico (Fig. 1), has demographic and epidemiological information systems that make viable the computing of ASYLL attributable to overweight and obesity.

The aim of this study was to estimate, by means of YLL and ASYLL, the burden of overweight and obesity among adults aged 20 years and older living in the state of Colima (Mexico). In addition, the leading causes of premature death were ranked and gender differences were evaluated.

## Methods

### Study design

A cross-sectional study took place from October 2015 to January 2016 using data from the Statistical and Epidemiological Death Registration System from the state of Colima. This surveillance system integrates data from issued death certificates, and its function is regulated by specific governmental normative lineaments (12). The events (deaths) that occurred between January 2010 and December 2014 were analyzed.

### Study population

Individuals aged 20 years and older at death with a registered basic cause of death of interest were enrolled. Thirteen underlying causes of death (International Classification of Diseases – 10th revision, ICD-10) were included: type 2 diabetes mellitus (E11.0–E11.9), arterial hypertension (I10), congestive heart failure (I50.0–I50.9), pulmonary embolism (I26.0 and I26.9), acute myocardial infarction (I21.0–I21.9), and malignant tumors (breast among postmenopausal women, C50.0–C50.9; colorectal, C18.0–C18.9; endometrial, C54.0–C54.9; esophageal, C15.0–C15.9; renal, C64; ovarian, C56; pancreatic, C25.0–C25.9; prostatic, C61). Because increased body weight is inversely associated with breast cancer risk before menopause (13), only deaths secondary to malignant tumors from the breast among postmenopausal women were analyzed. Females aged 48.1 years and older at death were considered as postmenopausal according to the mean age of menopause among Mexican women (14).

### Relative risks

In this study, the relative risks (RRs), sex-stratified, estimated by a previously published meta-analysis were used; this meta-analysis evaluated the association of overweight and obesity with the events of interest (15).

### Statistical analysis

Next, we calculated the burden of disease attributable to overweight and obesity. First, the population attributable fraction (PAF) (16) was estimated for each event as a function of the corresponding RR, and the prevalence of exposure among Mexican adults (≥20 years old) residing in the state of Colima evidenced by the Health and Nutrition National Survey 2012 (males, overweight 37.0%, obesity 30.5%; females, overweight 29.4%, obesity 42.7%). The PAF quantifies the proportional reduction in mortality that would potentially be observed with risk factor (overweight or obesity) reduction. Second, total age and cause-specific YLL were estimated following the GBD (Global Burden of Diseases, Injuries, and Risk Factors Study) procedure (17); standard life expectancy by each interval (_n_$e_{x}^{s}$) was obtained from the Tables of Life 2013 (Global Health Observatory) corresponding to Mexico (18). The arithmetic mean of age at death at each interval (_n_a_x_) was calculated using the whole registers from the analyzed database. The next parameters were fixed: discount rate (r)=0.03, age-weighting (β)=0.04, adjustment constant for age weights (C)=0.1658, and age-weighting modulation (K)=0. Templates (Microsoft^®^ Excel^®^) from the GBD study were used to compute the YLL, and the summary statistics were estimated using Stata^®^ MP 13.0 (StataCorp LP). Finally, the ASYLL rates per 10,000 inhabitants were estimated using the World Standard Population (2000–2025) (19).

### Ethical considerations

This study was approved by the Local Research and Ethics on Health Research Committee. Data regarding identification of individuals included in the study sample were omitted in order to preserve their anonymity.

## Results

Underlying causes of death of interest were registered on death certificates from 13,960 individuals. Data from 6,054 participants, for which death was attributable to overweight or obesity, were analyzed. Table 1 shows the number of deaths attributable to overweight or obesity by sex and age interval. Female proportion in the study sample was 46.6% (*n*=2,824), and the mean age at death was 55.1±21.3 years in both males and females.

**Table 1:** Population and study sample, Mexico 2010–2014

	Males				Females			
		
Age	_n_N_x_	_n_ $e_{x}^{s}$	_n_D_x_	_n_a_x_	_n_N_x_	_n_ $e_{x}^{s}$	_n_D_x_	_n_a_x_
20–24	29,487	59.5	18	22.3	30,583	54.4	2	22.0
25–29	25,994	54.6	24	27.2	27,142	49.9	3	27.2
30–34	24,590	49.8	30	32.4	25,931	45.4	12	32.2
35–39	23,868	45.0	58	37.6	24,995	41.0	16	37.5
40–44	20,101	40.2	82	42.0	21,331	36.7	42	42.3
45–49	17,505	35.5	114	47.8	18,307	32.3	85	47.7
50–54	15,247	31.0	184	52.3	15,670	28.1	152	52.5
55–59	11,581	26.6	272	57.6	11,720	24.0	171	57.5
60–64	8,939	22.5	327	62.5	9,416	20.2	246	62.5
65–69	6,765	18.7	367	67.6	7,041	16.6	307	67.6
70–74	4,918	15.1	393	72.4	5,321	13.4	367	72.7
75–79	3,375	11.9	399	77.6	3,601	10.6	333	77.5
80–84	2,050	9.1	320	82.0	2,515	8.3	305	82.0
≥85	2,176	6.8	642	90.1	2,611	6.3	783	90.3
Total	196,596		3,230		206,184		2,824	

*Symbols and sources*: _n_N_x_, population within each age group in the state of Colima, Mexico (Census of Population and Housing 2010, National Institute of Statistics and Geography); _n_$e_{x}^{s}$, standard life expectancy at each age interval (Tables of Life 2013: Mexico, Global Health Observatory); _n_D_x_, number of deaths secondary to specific causes of interest (Statistical and Epidemiological Death Registration System 2010–2014, Health Secretariat); _n_a_x_, arithmetic mean of age at death by age interval (Statistical and Epidemiological Death Registration System 2010–2014, Health Secretariat).

The overall burden of disease attributable to overweight and obesity was 36,086.9 YLL (Table 2). Among males, the estimated YLL due to overweight and obesity were 6,756.4 and 11,259.3, respectively. The number of YLL, among females, attributable to overweight and obesity were 6,756.4 and 11,259.3, respectively. In age-stratified analyses, YLL rate per 10,000 persons was higher in males than in females (overweight, 345.3 vs. 308.2 per 10,000 persons; obesity, 571.0 vs. 568.2 per 10,000 persons).

**Table 2:** YLL lost attributable to overweight and obesity and rate per 10,000 persons, Mexico 2010–2014

	Overweight		Obesity		Overall	
			
Age	YLL/rate		YLL/rate		YLL/rate	
*Males*						
20–24	0.0	0.0	32.9	11.2	32.9	11.2
25–29	53.7	20.7	80.6	31.0	134.3	51.7
30–34	103.4	42.1	181.0	73.6	284.4	115.6
35–39	177.4	74.3	297.3	124.6	474.7	198.9
40–44	259.1	128.9	444.2	221.0	703.3	349.9
45–49	421.1	240.6	722.2	412.5	1,143.3	653.1
50–54	592.8	388.8	990.6	649.7	1,583.4	1,038.4
55–59	893.2	771.3	1,579.0	1,363.5	2,472.2	2,134.8
60–64	1,004.2	1,123.3	1,752.0	1,960.0	2,756.2	3,083.3
65–69	1,002.1	1,481.4	1,735.0	2,564.7	2,737.1	4,046.0
70–74	844.6	1,717.4	1,470.8	2,990.6	2,315.4	4,708.0
75–79	614.4	1,820.4	994.8	2,944.7	1,609.2	4,765.1
80–84	346.9	1,692.0	494.7	2,413.3	841.6	4,105.3
≥85	443.5	2,038.3	484.2	2,225.4	927.7	4,263.7
Total	6,756.4	345.3	11,259.3	571.0	18,015.7	916.3
*Females*						
20–24	0.0	0.0	26.8	8.8	26.8	8.8
25–29	0.0	0.0	25.9	9.5	25.9	9.5
30–34	49.6	19.1	99.2	38.2	148.8	57.4
35–39	55.0	22.0	92.8	37.1	147.8	59.1
40–44	181.7	85.2	366.4	171.7	548.1	256.9
45–49	315.4	172.3	647.1	353.5	962.5	525.8
50–54	558.4	356.4	975.0	622.3	1,533.4	978.7
55–59	661.4	564.4	1,167.4	996.0	1,828.8	1,560.4
60–64	922.7	980.0	1,577.8	1,675.6	2,500.5	2,655.6
65–69	947.9	1,346.3	1,675.7	2,379.9	2,623.6	3,726.2
70–74	912.1	1,714.1	1,686.5	3,169.5	2,598.6	4,883.6
75–79	655.9	1,821.5	1,188.2	3,299.8	1,844.1	5,121.3
80–84	470.2	1,869.5	862.5	3,429.3	1,332.7	5,298.7
≥85	625.0	2,393.8	1,324.6	5,073.2	1,949.6	7,467.0
Total	6,355.3	308.2	11,715.9	568.2	18,071.2	876.5

YLL, years of life lost.

The leading causes of premature death are presented in Table 3. Type 2 diabetes mellitus represented the main cause of premature death (968.0 and 771.7 ASYLL per 10,000 persons in males and females, respectively) followed by acute myocardial infarction (males, 53.3 ASYLL; females, 84.3 ASYLL) and arterial hypertension (males, 20.1 ASYLL; females, 77.6 ASYLL). The overall estimated burden secondary to malignant tumors was 27.1 ASYLL and 66.2 ASYLL in males and females, respectively. Colorectal cancer had the highest site-specific burden, mainly in females (15.9 vs. 11.9 ASYLL in males).

**Table 3:** ASYLL by cause of death, Mexico 2010–2014

		YLL			
			
Event		*n*	%	Rate^a^	ASYLL^b^
*Males*					
1	Type 2 diabetes mellitus	15,815.3	87.8	804.5	968.0
2	Acute myocardial infarction	1,245.1	6.9	63.3	53.5
	*All cancers*^c^	443.1	2.5	22.5	27.1
3	Arterial hypertension	332.0	1.8	16.9	20.1
4	Colorectal cancer	186.8	1.0	9.5	11.9
5	Congestive heart failure	128.1	0.7	6.5	7.4
6	Prostatic cancer	103.4	0.6	5.3	6.4
7	Pancreatic cancer	94.0	0.5	4.8	6.1
8	Pulmonary embolism	52.1	0.3	2.7	3.1
9	Renal cancer	46.8	0.3	2.4	2.6
10	Esophageal cancer	12.1	0.1	0.6	0.8
	Total	18,015.7		916.3	1,098.4
*Females*					
1	Type 2 diabetes mellitus	13,302.2	73.6	645.2	771.7
2	Acute myocardial infarction	1,584.7	8.8	76.9	84.3
3	Arterial hypertension	1,481.0	8.2	71.8	77.6
	*All cancers*^c^	1,141.2	6.3	55.3	66.2
4	Congestive heart failure	419.7	2.3	20.4	21.9
5	Colorectal cancer	275.9	1.5	13.4	15.9
6	Endometrial cancer	183.6	1.0	8.9	10.9
7	Ovarian cancer	187.2	1.0	9.1	10.2
8	Breast cancer	173.4	1.0	8.4	9.9
9	Renal cancer	160.8	0.9	7.8	9.4
10	Pancreatic cancer	154.6	0.9	7.5	8.8
11	Pulmonary embolism	142.4	0.8	6.9	7.7
12	Esophageal cancer	5.7	0.03	0.3	0.2
	Total	18,071.2		876.5	1,028.5

YLL, years of life lost; ASYLL, age-standardized expected YLL.

a Per 10,000 inhabitants

b Rates per 10,000 inhabitants were estimated using the World Standard Population (2000–2025)

c Includes breast cancer among postmenopausal women and colorectal, endometrial, esophageal, renal, ovarian, pancreatic, and prostatic cancer.

## Discussion

Our findings suggest that, in the state of Colima (Mexico) from 2010 to 2014 and measuring 13 specific causes of death, the attributable burden of overweight and obesity was 18,015.7 and 18,071.2 YLL in adult males and females, respectively. The YLL for all deaths in the study period are 148,573.8 in males and 87,615.3 in females. The overall proportion of YLL attributable to the exposure to overweight and obesity is 12.1 and 20.6% in males and females, respectively. Therefore, among adults ≥20 years old, an average of 7,200 YLL may be prevented annually by the implementation of health policies focusing on the reduction of exposure to overweight and obesity.

Chronic degenerative diseases were the main cause of premature death; the aggregated mortality of type 2 diabetes mellitus (29,117.5 YLL), acute myocardial infarction (2,829.8 YLL), and arterial hypertension (1,813.0 YLL) represents more than 93% of total YLL from the study sample. Overweight and obesity are major risk factors for these diseases (15).

The World Health Organization (WHO) definition of premature death includes deaths under 70 years old (20). In this study, the standard life expectancy by age interval was used and enabled us to estimate the YLL and other parameters of interest among individuals aged 70 years and older at death.

Type 2 diabetes mellitus was the non-communicable disease with the highest burden; in both sexes, more than 70% of ASYLL were secondary to this metabolic disease. Similar findings were previously observed in other populations (8). Epidemic characteristics of type 2 diabetes mellitus have been observed in Mexico; according to the Health and Nutrition National Survey 2012, its prevalence among adults aged 20 years and older is 9.2%. The estimated prevalence in the state of Colima is 9.1 and 9.9% in males and females, respectively (21).

The burden of type 2 diabetes mellitus is increased by the substantial costs of medical attention of these patients. A recently published analysis evidenced that the annual cost per diabetic patient ranges from US$699 to US$748 (22). Direct costs of this chronic disease represent approximately 14% of total health spending (23) and, since 2000, diabetes mellitus is the leading cause of death in Mexico (13.8% of deaths) (5).

Worldwide, ischemic heart disease, as acute myocardial infarction, is the principal cause of death (24). However, deaths attributable to cardiovascular diseases may be underestimated by the use of ‘garbage’ codes in death certificates that do not correctly identify the main cause of death (25).

In addition to overweight and obesity, a high prevalence of other known cardiovascular risk factors such as tobacco use (31.9%) and hypercholesterolemia (13.8%) have been documented in the Mexican adult population (26); type 2 diabetes mellitus is also associated with an increased risk of cardiovascular diseases (27). Age-adjusted incidence of myocardial infarction in this population is 6.6 and 4.8 per 1,000 person-years (28).

A high proportion (31.5%) of Mexican adults have arterial hypertension and almost half of them (47.3%) are unaware of their disease (29). Only 56.8% of hypertension patients have a good control of blood pressure levels (<140/90 mmHg) (30).

Chronic non-communicable disease associated with overweight and obesity represents a major challenge for health systems. Body weight reduction reverses the excess risk of type 2 diabetes mellitus (31, 32) and cardiovascular diseases (33). The promotion of healthy lifestyles including a balanced diet, restricted caloric intake, and regular physical activity is priority in order to prevent these events (34). In addition, steady preventive actions at cluster and individual level are needed because frequent body weight fluctuations have been associated with an increased risk of chronic diseases and mortality rate (35, 36).

Despite the efforts in prevention, early detection, and opportune and accurate medical treatment, cancer morbidity and mortality have increased because of population aging and high prevalence of known major risk factors such as smoking and obesity (37). Malignant tumors are an important cause of premature death in adults residing in the state of Colima; in the study period, the overall burden estimate secondary to malignant neoplasms was 11,184.0 YLL. The excess risk fraction attributable to overweight and obesity was 14.2% (1,584.3 YLL).

Malignant neoplasms are among the top five causes of disease burden in Mexico, mainly secondary to tumors from breast, prostate, respiratory tract (trachea, bronchi and lung), gastrointestinal track (stomach and colorectal), and leukemia (38). Overweight and obesity are associated with increased risk of breast, prostate, and colorectal cancer and were analyzed in this study (39–41).

Our findings suggest that, among malignant tumors, colorectal cancer has the highest burden. The attributable fraction of overweight and obesity was also high in males (52.5%) and females (31.2%). This finding correlates with a previously published study that documented an increasing incidence of colorectal cancer and related mortality among Mexican individuals (42).

In females, the overall burden of disease of postmenopausal breast cancer and ovarian cancer was the highest in the study period (ovarian, 187.2 YLL; breast, 173.4 YLL), but only 6.1 and 13.6% of risk excess were attributable to a high BMI. Therefore, research regarding modifiable risk factors such as physical activity is needed in order to reduce cancer-related mortality (43–45).

Regarding malignant prostate tumors, the disease burden in the study period was 2,006.0 YLL, and 103.4 YLL (5.1%) were attributable to a high BMI. In Mexico, 80% of prostate malignant tumors are diagnosed in advanced clinical stages and have a poor prognosis (46).

The mechanisms of obesity-induced cancer risk increase are not completely understood (47). Recently, a role of increased levels of placental growth factor in obesity-induced tumor progression has been suggested (6). Targeting overweight and obesity may improve cancer prevention and outcomes after diagnosis (48). Therefore, a cancer burden reduction may be potentially observed.

YLL and years lived with disability (YLD), combined, inform disability-adjusted life-years (DALY). However, data regarding YLD are not collected systematically by the surveillance system (12). The computing of YLD is not useful for relatively small regions, and ASYLL represent a good analytical tool to measure the burden of disease (11).

Reliability of causes of death registered on death certificates is a fundamental aspect of this research. According to estimates, the Mexican system of death registration is one of the best worldwide in terms of quality and integrity of data (49). Concordance between the causes of death in medical death certificates issued in Mexico, when compared versus the gold standard, is 66.5% among adults. The accuracy of registered underlying cause of death in this age group is 0.780 (50).

## Conclusion

Our findings provide quantitative evidence at regional level of burden of disease attributable to overweight and obesity among a Mexican adult population. Overweight and obesity are major and potentially modifiable risk factors for type 2 diabetes mellitus and cardiovascular diseases that are main causes of premature death in the study population.

This work exposes the attributable fraction of obesity. The number of healthy (disability-free) ASYLL is an alternative that could be developed and used by policymakers and health professionals to highlight the total risk of obesity. Computing disability-adjusted life-years needs more information than calculating YLL, because subjective decisions would be required to determine what constitutes a disability and to develop severity-of-disability ratings. However, the YLL metric provides a useful strategy to illustrate the most tangible cost of obesity and overweight.

## Paper context

The exposure to overweight and obesity is a major risk factor for non-communicable diseases associated with a high mortality among adult individuals. The contribution of both exposures to premature death, at regional-level, was quantified in this study. Strategies for preventing overweight and obesity may decrease the incidence and mortality associated with these diseases.
